# Assessing the Surgical Efficiency of Instrument Miniaturization: Comparison of Holmium Laser Enucleation of the Prostate (HoLEP) and Minimally Invasive Laser Enucleation of the Prostate (MiLEP)

**DOI:** 10.7759/cureus.104201

**Published:** 2026-02-24

**Authors:** Francisco Gomez-Regalado, Leonardo Ruiz-Guerrero, Said Castro-Zazueta, María Luisa Vazquez-Villegas, Carlos Tejeda-Andrade, Alejandro Figueroa-Garcia, Mario Eduardo Galland-Novelo

**Affiliations:** 1 Urology, Hospital Angeles Del Carmen, Guadalajara, MEX; 2 Epidemiology, Unidad Medica Familiar 4, Instituto Mexicano del Seguro Social, Guadalajara, MEX

**Keywords:** anatomic endoscopic enucleation of prostate, benign prostate hyperplasia, holep, milep, miniaturized instrumentation, prostate

## Abstract

Introduction: Benign prostatic hyperplasia is one of the most common medical problems. It can be treated with holmium laser enucleation of the prostate (HoLEP), which provides efficacy with few complications and a short hospitalization. A more recent evolution of this technique is minimally invasive laser enucleation of the prostate (MiLEP), using smaller sheaths, which aims to further reduce urethral trauma while maintaining surgical efficiency.

Objective: This study set out to compare HoLEP and MiLEP interventions in prostate enucleation to assess the impact of instrument miniaturization on surgical efficiency.

Methods: In this observational study, we analyzed patients undergoing prostate enucleation in the Minimally Invasive Urology Service at Hospital Angeles del Carmen. Patients were recruited from March 1 to July 31, 2025. We included patients with a prostate volume ≤ 80 cc, International Prostate Symptom Score (IPSS) value >8, and an American Society of Anesthesiologists (ASA) Staging of I or II, and excluded those with a history of bladder pathology, prior urinary catheterization, or urolithiasis. Epidemiological and clinical variables and surgical intervention were evaluated. Statistical analysis was descriptive, and medians were compared with the Mann-Whitney U test.

Results: A total of 40 patients were included, and the HoLEP and MiLEP techniques were compared. The median age was 68 years, IPSS 21, and prostate tissue 24 gr. Enucleation time was longer in the subgroup of HoLEP (p=0.01). Surgical time was shorter in the subgroup of MiLEP (p=0.01). No differences were observed in morcellation time, enucleated tissue weight, enucleation efficiency, and morcellation efficiency. A trend toward shorter hemostasis time was observed in MiLEP, though this trend did not achieve statistical significance.

Conclusion: Miniaturized instrumentation using a 22 Fr sheath in MiLEP is comparable to the standard 26 Fr sheath in HoLEP in terms of surgical efficiency and safety. The smaller sheath may provide additional advantages. Further studies with longer follow-up are needed to evaluate the long-term functional outcomes.

## Introduction

Benign prostatic hyperplasia (BPH) is a condition that predominantly affects older men; it is estimated that 50% of men aged 60 years have this condition [1]. The first minimally invasive treatment was transurethral resection of the prostate (TURP), which for decades was considered the gold standard after its advantages over open surgery were demonstrated [2]. However, in the 1990s, Gilling and Fraundorfer developed a prostate enucleation technique that employed the ablative power of the holmium laser. This novel technique, termed holmium laser enucleation of the prostate (HoLEP), required a high-power holmium laser and a morcellation system [3]. HoLEP offers a similar operative duration and functional outcomes to open prostatectomy, while providing fewer perioperative complications and shorter hospital stays compared with TURP [4,5].

The standard HoLEP equipment uses a 26 Fr resectoscope sheath [1]. Since its introduction, enucleation techniques have evolved with increasing surgical experience and patient outcomes, beginning with the original trilobar technique described in 1998 by Peter Gilling [6]. This approach involves separate enucleation of the lateral and median lobes, using three bladder neck incisions (BNIs). Over time, the bilobar technique was developed, eliminating one bladder neck incision, as well as reducing the bleeding time and avoiding lateral plane crossover. More recently, the en-bloc technique has emerged, requiring a single bladder neck incision to further decrease operative time and bleeding [6,7].

It is important to note that all current techniques require early release of the sphincter mucosa as an initial step to minimize trauma and reduce postoperative urinary incontinence [7,8]. HoLEP provides advantages such as the use of minimally invasive instruments regardless of the prostate size or patient comorbidities, along with a reduced blood loss and shorter hospital stays when compared with traditional open and endoscopic treatments [8,9]. To build on those advantages, Figueiredo introduced the concept of minimally invasive laser enucleation of the prostate (MiLEP) using a 22 Fr sheath, with the goal of reducing urethral trauma, minimizing perioperative morbidity, and enhancing postoperative recovery. The central premise is that these improvements may lead to fewer short- and long-term complications [10]. Additional reported advantages of MiLEP include improved thermal stability, reduced irrigation fluid volume, and less dilation compared with HoLEP [11]. 

Moreover, the advantages of MiLEP over HoLEP described in the literature include decreased urethral dilation resulting in fewer complications, without compromising the surgical efficiency. However, no differences have been demonstrated in the urinary catheter duration or length of hospitalization. Conversely, the use of a larger-diameter sheath, as in HoLEP, was reported to decrease morcellation time as a result of improved irrigation flow and visualization [12,13].

This study set out to assess the impact of instrument miniaturization on surgical efficiency by comparing HoLEP and MiLEP interventions in prostate enucleation.

## Materials and methods

This was an observational study conducted at the Hospital Angeles del Carmen, Guadalajara, Mexico. This center receives more than 200 cases per year with a diagnosis of benign prostatic hyperplasia, being part of the private healthcare sector. The study was approved by the Research Ethics Committee of the hospital.

Study population

Patients undergoing prostate enucleation in the Minimally Invasive Urology Service between March 1, 2025, and July 31, 2025, were considered for the study.

Patients with a diagnosis of prostatic enlargement on pelvic transrectal ultrasound with a prostate volume ≤ 80 cc, on the basis of the criteria for International Prostate Symptom Score (IPSS) of >8 [14], and an American Society of Anesthesiologists (ASA) physical status classification of I or II [15] were included. The decision to include patients with a prostate volume <80 cc was based on the observation that morcellation in larger prostates is frequently associated with impaired visualization and may require the use of a larger-caliber morcelloscope, thereby offsetting the advantage of a smaller-diameter instrument. In addition, de Figueiredo and Teloken recommend and report that their group prefers the use of ultra-slim sheaths in patients with prostate volumes <80 cc to improve visualization [10]. Exclusion criteria were a bladder pathology history, prior urinary catheterization, or urolithiasis.

It is important to mention that all the patients were on current alpha-blocker therapy prior to the surgery, and no patient had prior prostate instrumentation. 

Procedure

Patients were divided into the HoLEP or MiLEP intervention group following a consecutive assignment approach, in which one patient was allocated to the HoLEP group and the next to the MiLEP group, continuing in this sequence. In the HoLEP group, a 26 Fr resectoscope sheath was used. Whereas in the MiLEP group, a 22 Fr sheath was employed. Both were coupled with a 30° lens.

The procedures were performed by two urologic surgeons with similar experience profiles. Both surgeons had previously conducted over 200 enucleation procedures with the same surgical technique using 26 Fr sheaths. One surgeon was assigned to the HoLEP group and the other to the MiLEP group, in a non-selective manner. These procedures were scheduled in the same hospital system. In all patients, the urinary catheter was removed after 36 hours postoperatively.

All procedures were performed using the Lumenis Pulse™ 120H laser system with MOSES™ 2.0 technology (Boston Scientific Corporation, Marlborough, Massachusetts, United States) with a 550-μm MOSES laser fiber (Boston Scientific Corporation). Enucleation was performed using energy settings of 2 J and 50 Hz, and coagulation at 1 J and 40 Hz. Enucleated prostatic tissue was morcellated using a Hawk morcellator under continuous irrigation with 0.9% normal saline.

All patients underwent combined anesthesia via a subarachnoid (spinal) block and intravenous sedation. The enucleation technique used in all cases was the en bloc, no-touch approach described by Scoffone and Cracco, which includes early apical release of the external urethral sphincter and dissection of the bladder mucosa [8]. Prophylactic antibiotics were administered in all cases, either as broad-spectrum agents or tailored to the preoperative urine culture and sensitivity results. 

Data collection

One trained researcher used a structured data-collection form to record enucleation time (minutes), morcellation time (minutes), enucleated tissue weight (grams), enucleation efficiency (g/min), morcellation efficiency (g/min), and hemostasis time (minutes).

Statistical analysis

The results for quantitative variables are reported as medians and ranges. Therefore, the Mann-Whitney U test was utilized for comparisons between the medians of quantitative variables of the two groups: HoLEP and MiLEP. The threshold of statistical significance was noted as p ≤0.05. IBM SPSS Statistics for Windows, version 20 (IBM Corp., Armonk, New York, United States) was used for statistical procedures.

## Results

A total of 40 patients were included in the study, with 20 in the HoLEP group and 20 in the MiLEP group. Table 1 summarizes the description of clinical features and surgical characteristics of the patients. Their median age was 68 years, the median IPSS was 21, the median surgical time was 41 minutes, and the median enucleated tissue weight was 24 gr.

**Table 1:** Description of clinical features and surgical characteristics of the patients (N=40) IPSS: International Prostate Symptom Score

Variables	Median (Range)
Age (years)	68 (48-82)
Prostate Volume (cc)	60 (30-80)
IPSS score	21 (14-32)
Enucleation time (minutes)	31 (14-68)
Morcellation time (minutes)	7 (2-30)
Hemostasis Time (minutes)	2 (0-22)
Obtained Prostate Tissue	24 (6-60)
Enucleation Efficiency	1 (0.2-2.2)
Morcelation Efficiency	3 (1.1-9.0)
Surgical Time (minutes)	41 (19-79)

Table 2 compares perioperative outcomes between the two groups, HoLEP and MiLEP. These groups were similar in age, prostate volume, IPSS score, morcellation time, hemostasis time, enucleated tissue weight, enucleation efficiency, and morcellation efficiency. Enucleation time was longer in the subgroup of HoLEP compared to MiLEP (p = 0.01), as was the surgical time (p = 0.01). 

**Table 2:** Comparison of clinical features and surgical characteristics between HoLEP versus MiLEP Medians were compared with the Mann-Whitney U test HoLEP: holmium laser enucleation of the prostate; MiLEP: minimally invasive laser enucleation of the prostate; IPSS: International Prostate Symptom Score

Variables	HoLEP (n= 20), median (range)	MiLEP (n=20), median (range)	p-value
Age (years)	65 (48-82)	69 (51-80)	0.20
Prostate Volume (cc)	60 (40-80)	54 (30-80)	0.12
IPSS score	21 (14-25)	22 (14-32)	0.13
Enucleation time (minutes)	36 (17-68)	28 (14-44)	0.01
Morcellation time (minutes)	8 (3-20)	6 (2-30)	0.09
Hemostasis time (minutes)	6 (0-22)	0 (0-18)	0.07
Obtained Prostate Tissue	26 (7-60)	22 (6-50)	0.44
Enucleation Efficiency	1 (0.2-1.6)	1 (0.3-2.2)	0.50
Morcellation Efficiency	3 (1-9)	3 (1.6-8)	0.60
Surgical time (minutes)	47 (26-79)	39 (19-70)	0.01

All patients had a one-day hospital stay, with discharge on the day after urinary catheter removal. None of the patients experienced either major or minor complications during the intraoperative period or their hospitalization.

## Discussion

In this study, we observed that HoLEP required more time for enucleation and the surgical process compared with MiLEP. No differences were observed in morcellation time, enucleated tissue weight, enucleation efficiency, and morcellation efficiency; although a trend toward shorter hemostasis time was observed in MiLEP, this did not achieve statistical significance.

A retrospective study conducted by Schmidt et al. compared similar techniques and showed a difference in coagulation time (p = 0.02) and morcellation time (p = 0.03) between HoLEP and MiLEP, with HoLEP demonstrating a more efficient coagulation time, while MiLEP had a shorter morcellation time [12]. In comparison, our study demonstrated a significant reduction in enucleation time in the MiLEP group, which was accompanied by a corresponding decrease in overall operative time. Additionally, our patients were younger and had smaller prostate volumes.

Alaradi et al. evaluated 90 patients in a randomized controlled trial [16], which compared the same surgical procedures as our study. They observed acceptable visibility during the enucleation step, which allowed visualization of the prostate tissue. Furthermore, their results showed that the 26 Fr sheath reduced the catheterization time, with that result marking a difference from our study.

The study by Ramadhani et al. compared the use of a Moses technology laser with standard holmium for prostate enucleation. The authors concluded that the Moses laser achieved reduced hemostasis and enucleation times [1]. In comparison with the Moses group in the previously mentioned study, our HoLEP cohort demonstrated similar hemostasis outcomes; however, the use of MiLEP was associated with a shorter hemostasis time. On the other hand, Günes et al. described no difference in resection time and resected prostate tissue weight between the 24 and 26 Fr sheaths [17], a similar result to that in our study.

Within the current body of evidence, this study provides a focused, head-to-head comparison of HoLEP and MiLEP performed under uniform institutional conditions. Isolating individual operative steps, it helps delineate which aspects of the procedure are influenced by technique and which remain largely unaffected. To date, there is limited evidence directly comparing the surgical efficiency of HoLEP and MiLEP for prostate enucleation. To the best of our knowledge, this is the first study to demonstrate a reduction in enucleation time and overall surgical time in the MiLEP group.

Our study had a key limitation that must be taken into account. These patients were treated at a single center, and the outcomes might reflect only the skill of one surgical group. To overcome this, a multicenter study including patients and surgical groups from other regions should be considered.

## Conclusions

In this study, the use of smaller-diameter sheaths (22 Fr) in MiLEP procedures was observed to achieve outcomes comparable to those obtained with standard HoLEP sheaths (26 Fr), with respect to enucleation efficiency, morcellation efficiency, and hemostasis time. Smaller sheaths offer advantages such as reduced mucosal trauma, better temperature maintenance, and decreased irrigation fluid absorption, which may translate into improved patient safety. In our evaluation, we found no significant difference in surgical efficiency when using a smaller-caliber instrument; however, future assessment of complications is necessary to determine whether it offers any advantage over standard-sized equipment. To extend our knowledge, further prospective multicentric studies with extended follow-up periods are warranted to evaluate long-term complications such as urethral strictures and changes in continence status.
